# Leaf area and pubescence drive sedimentation on leaf surfaces during flooding

**DOI:** 10.1007/s00442-020-04664-2

**Published:** 2020-05-17

**Authors:** Lena Kretz, Carolin Seele, Fons van der Plas, Alexandra Weigelt, Christian Wirth

**Affiliations:** 1 Systematic Botany and Functional Biodiversity, Life science, Leipzig University, Leipzig, Germany; 2 German Centre for Integrative Biodiversity Research (iDiv) Halle-Jena-Leipzig, Leipzig, Germany; 3 Max Planck Institute for Biogeochemistry, Jena, Germany

**Keywords:** Functional traits, Flume experiment, Floodplain, Sediment retention

## Abstract

Worldwide, stream water is increasingly loaded with sediments and nutrients, due to processes such as accelerated soil erosion and overfertilization caused by agricultural intensification. This leads to increases in eutrophication and silting up of bottom sediments. Floodplains can play an important role in mitigating these problems, by removing sediment from rivers via water filtration and retention. Fine sediment is accumulated on the soil in between plants as well as on plant surfaces. However, it is still poorly understood how plant species facilitate leaf surface sedimentation via their leaf traits. In a flume experiment, we investigated to what extent the leaf traits (area, length, perimeter, pinnation, pubescence, surface roughness, flexibility and wettability) influence leaf surface sedimentation. We exposed leaves of 30 plant species to an artificial flood, and measured the fine sediment load the leaves captured after 24 h. Our results show that leaf traits overall explain 65% of the variation of fine sedimentation on leaves. Especially adaxial pubescence and leaf area strongly drove sedimentation. Hairy leaves accumulate more sediment per leaf area, presumably, because hairs create a buffer zone of reduced flow velocity which enhances sedimentation between the hairs. Additionally, for leaves with no or few hairs, sedimentation decreased with increasing leaf area, because most likely the more turbulent boundary layer of larger leaves allows less sediment to settle. Our results provide a first understanding of how plants can be selected based on their leaf traits for maximizing the sediment retention on floodplains, thereby providing a key ecosystem service.

**Electronic supplementary material:**

The online version of this article (10.1007/s00442-020-04664-2) contains supplementary material, which is available to authorized users.

## Introduction

Worldwide, sediment and nutrient loads in stream water are increasing due to anthropogenic activities (Sharma and Rai 2004; Quilbé et al. 2006; Hunter and Walton 2008; Jones et al. 2012). Industrial agriculture and forestry, but also sealing of soil (e.g. covering of soil with buildings or roads) and mining cause overfertilization of soils, soil erosion and surface water runoff that are jointly responsible for increases in the sediment and nutrient load of streams (Turnpenny and Williams 1980; Carpenter et al. 1998; Hancock 2002; Grizzetti et al. 2008; Bernhardt and Palmer 2011; Berendse et al. 2015). Consequences are eutrophication of the stream water and silting up of sediment in branches and the mouth of the stream (Bouwman et al. 2013; Habersack et al. 2016). Under natural conditions, floods counteract these processes by depositing sediment particles and nutrients from streams into floodplains, which function as a sink for both (Naiman and Décamps 1997; Asselman et al. 2003; Walling et al. 2003; Taylor et al. 2008; Bouwman et al. 2013). Thereby, floodplains provide key ecosystem services of sediment and nutrient retention and water filtration (Hopkins et al. 2018; Conte et al. 2011). However, river straightening and embankment have dramatically reduced floodplain area, so that floodplains count worldwide as one of the most threatened ecosystems (Naiman and Décamps 1997; Tockner and Stanford 2002; Thoms 2003; Steiger et al. 2005). To restore the ecosystem service of water filtration, many countries have launched programs to reactivate former floodplains. In addition, existing floodplain areas could be managed to maximise retention capacities during overbank flow conditions. To achieve this, we need to improve our understanding of how plant and vegetation characteristics enhance sedimentation.

It is still unknown if and how functional and structural diversity of floodplain vegetation enhances retention and water filtration. Sediment retention is a complex phenomenon that depends on different biogeomorphic processes in the floodplain (Corenblit et al. 2011). While coarse sedimentation is mostly influenced by the geomorphology of the floodplain, the vegetation type and structure are most relevant for fine sedimentation (Corenblit et al. 2011; Manners et al. 2013). Communities of herbaceous pioneer vegetation are more efficient in accumulating fine sediments compared to shrublands and floodplain forests (Corenblit et al. 2009). Furthermore, the ability of plant communities to accumulate sediments might increase with increasing diversity and associated functional and structural complexity of the plant communities (Emerson and Kolm 2005). Various studies have shown that vegetation acts as a sediment filter causing sedimentation between the plants and on the plant surfaces; however, none of these studies focused on species identity and diversity effects (Karr and Schlosser 1978; Blanco-Canqui et al. 2004; Pan et al. 2011; Gurnell et al. 2012; Kervroëdan et al. 2018). Elliott (2000) emphasized the importance of sedimentation on plant surfaces in addition to the vertical structural complexity of the plant. Instream stands of macrophytes slow down flow velocity and reduce turbulence within the stand, which causes accumulation of fine sediment (Sand-Jensen 1998; Clarke 2002; James et al. 2002; Palmer et al. 2004; Ortiz et al. 2013). On-plant sedimentation is low for macrophytes, since they have adapted leaves that streamline with the water flow (Jones et al. 2012; Sand-Jensen 1998; Rovira et al. 2016). Herbaceous floodplain species, however, are not well adapted to inundation, so the on-plant sedimentation may play an important role for sediment retention (Elliott 2000).

To our knowledge, there is currently no study that investigated the on-plant sedimentation of herbaceous floodplain vegetation after inundation and related it to plant leaf traits. Plant leaves vary in size, morphology and surface structure (Koch et al. 2009), and it is possible that these and other leaf traits determine sediment accumulation. Studies focusing on the instream vegetation have shown that for macrophytes flat, smooth and flexible leaves capture the least sediment in the surrounding (Sand-Jensen 1998; Jones et al. 2012; Rovira et al. 2016). Furthermore, a study using artificial leaves showed that shape, serration, roughness and flexibility of leaves have an impact on the drag force and turbulence intensity, which are expected to alter sedimentation (Albayrak et al. 2012). Also, studies on airborne particle deposition on leaf surfaces found trait effects on deposition rate. Since fluid dynamics are similar for water and air (except that water is more strongly affected by the viscosity of the fluid), studies on airborne deposition may also be informative for sedimentation on leaves under water. The deposition of airborne particles is strongly affected by leaf area, surface waxes (wettability), pubescence and surface roughness (Wedding et al. 1975; Little 1977; Burkhardt et al. 1995; Sæbø et al. 2012; Weber et al. 2014).

There are five main classes of leaf traits that likely determine the sedimentation on leaf surfaces. First, hair density on the leaf surface has a positive effect on airborne particle adhesion (Räsänen et al. 2013; Weber et al. 2014). Hairs act as obstacles to the flow, building a buffer zone of reduced flow velocity (Wedding et al. 1975). However, very dense hairs on the leaf surface can also cause a cleaning effect by enhancing water runoff (Otten and Herminghaus 2004) leading to reduced sedimentation. Second, the total leaf area may influence sedimentation, although negative effects as well as non-significant effects have been reported for studies on airborne particles (Sæbø et al. 2012; Räsänen et al. 2013; Weber et al. 2014). Flow dynamics of the boundary layer on the leaf surface cause greater turbulence with distance and, consequently, less sedimentation on the leaf surface. Third, flexible leaves streamline better with the flow, while stiff leaves cause greater near-surface turbulence (Horn and Richards 2007; Chen et al. 2011; Nepf 2012). Forth, the roughness of a leaf can increase sedimentation, which was shown for macrophytes (Jones et al. 2012) and for airborne particle deposition on terrestrial plant leaves (Sand-Jensen 1998; Weber et al. 2014). Fifth, wettability of leaves depends on various traits and mechanisms, including repellence due to wax layers (the lotus effect), pubescence (Otten and Herminghaus 2004; Koch et al. 2008; Bhushan et al. 2009) and the ultra-structure roughness (Bhushan et al. 2009; Wang et al. 2014). So far, increasing and decreasing effects of wettability on sedimentation were found (Neinhuis and Barthlott 1998; Räsänen et al. 2013). However, it is still unclear how all these mentioned leaf traits influence sedimentation on submerged leaf surfaces of herbaceous vegetation during overflow conditions.

Our aim is to comprehensively examine how leaf traits influence sedimentation in floodplains. We used flumes to experimentally simulate inundation of leaves of 30 species in sediment-rich water and to quantify the effect of traits on sediment accumulation on the leaf surface per unit of leaf area. We hypothesize that leaf surface sedimentation increases with decreasing area and length. Sedimentation may also increase with decreasing perimeter and pinnation, caused by reduced turbulence around the leaf. Furthermore, we expect sedimentation to be positively correlated to leaf pubescence and roughness, since both build buffer zones for sediment to settle. We further expect that sedimentation increases with increasing wettability due to increasing contact area and also increases with increasing flexibility due to reduced near-surface turbulence.

## Materials and methods

### Selection of the species

We selected herbaceous plant species to span the gradient of variation in the investigated leaf traits while ensuring that typical floodplain species were also well represented. This was done by categorizing plant leaves a priori using three factors with two to three levels (leaf flexibility: stiff vs flexible, roughness: rough vs smooth, pubescence: dense hairs vs sparse hairs vs no hairs, see Supp 1), and assigning candidate species to these. While the categorization was arbitrary, all categories had clear links to our traits of interest. We used plant community inventories of floodplain meadows along the Mulde River (51°43′–46′ N, 12°17′–18′ E) conducted in the context of the conservation project “Wilde Mulde” (Wilde Mulde—Revitalization of a wild river landscape in Central Germany) and selected 16 herbaceous species observed during these inventories (Supp 1). Then, we supplemented these with 14 additional species from the Botanical Garden of Leipzig to fill gaps in predictor trait space. For each combination of trait categories, we measured a minimum of two different species out of at least two different families. We did not find any species to fill the trait category combinations “stiff” and “dense hairs” with either “rough” or “smooth” species. All species were collected in the Botanical Garden of Leipzig and surroundings, which ensured that we could use fresh plant material for the experiment.

### Experimental set-up

**Fig. 1:**
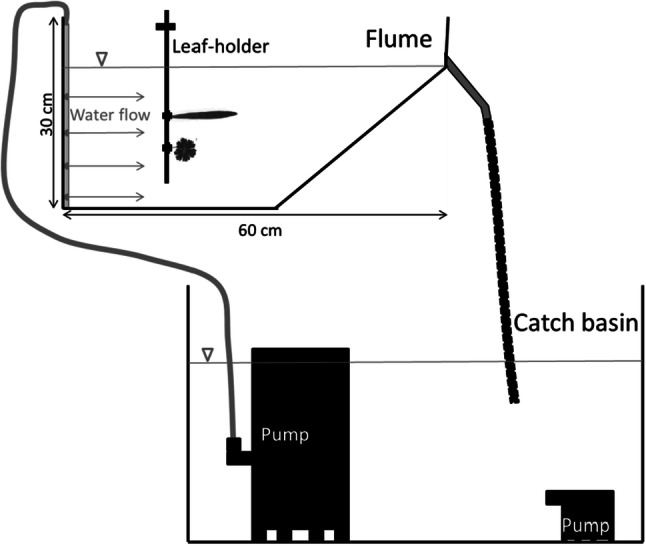
Sketch of the experimental setup. Experimental flume with the water flowing from left to right, overflows into the catch basin and is pumped up into the inflow of the flume. We fixed the leaves with clips to the leaf-holder within the flume

We performed the flume experiment in the greenhouse of the Botanical Garden in Leipzig. The eight flumes were self-made with modified standard aquariums (30 × 30 × 50 cm^3^, Fig. 1). We used handcraft clay as sediment (Ø < 2 µm, “Soft-Ton”, Glorex), since fine particles are most relevant regarding nutrient bounding (Naiman and Décamps 1997), and larger particles could not stay in a constant solution for longer. A rainwater pump (with power of 400 W, Tauchpumpe 400, CMI) generated the water flow. Additionally, we placed a second small aquarium pump in the catch basin to avoid sedimentation on its bottom (compactON 1000, EHEIM). Four tubes with four small effluences each distributed the flow in the aquarium unidirectional and as even as possible to simulate natural overbank conditions on a meadow. Nevertheless, there were some reflux and some smaller turbulence in the flume caused by the skewed glass plate at the outlet. The mean flow rate was 13.8 L min^−1^ with mean velocity at the leaf-holder of 5.6 cm s^−1^. The leaf-holder fixed the leaves on a frame with small clips in the middle of the flume. It consisted of three rods beside each other, each with two clips, resulting in six potential positions to fix single leaves (Fig. 1). We cleaned the whole flume set-up every week to avoid algae growth and to keep the amounts of water and sediment in the flume constant. Each week, we solved 38 g dry clay in 60 L water per flume to create a saturated solution.

### Sample processing

Of each sampled species, we picked eight leaves and kept them in a moist plastic bag to avoid desiccation during transport. Before fixing the leaves into the flume, we scanned each leaf (Expression 11000XL, Epson) to record the leaf form (Supp 2). To avoid interference between the leaves, we restricted the number of leaves per flume between one and a maximum of six, depending on the size of the leaves. Each run lasted 24 h, and we ran eight flumes simultaneously. Each flume contained one leaf per species. After 24 h, we took leaves carefully out of the flume, washed off accumulated sediment from both sides into a small beaker with 40 mL water, and carefully cleaned them with a soft toothbrush. We filled additional beakers with 40 mL water of each flume to control for variation in sediment solution per flume and day in subsequent analyses. To quantify the sediment mass, we dried the beakers with solution in a dry oven at 70 °C, removed the sediment, dried the sediment sample again until constant final weight and weighed it afterwards.

We performed a second set of runs to control for the effects of leaf shape and size. For this second experiment, we cut the leaves to a standardized size of 2 × 6 cm^2^ with a scalpel. We chose this shape and area to get a standardized leaf area and to include as many species as possible. We included 12 species out of the species set (Supp 1). All other procedures and measurements were done in the same way as with the entire leaves.

### Trait measurements

We sampled ten additional leaves per species used in the flume experiment to measure the following leaf traits: pubescence, roughness, flexibility (few species just have five replicates) and wettability (detailed measurements are listed in Supp 3). All leaves of the sampled species were collected freshly in the vicinity. We used the whole leaf in the flume for all species except for *Onobrychis viciifolia* where we treated single leaflets as leaves. For measuring the pubescence, we took microscopic photos with an AxioVision SE64 Rel. 4.9 (Zeiss). On an image of approximately 1 cm^2^, we counted the hairs manually using the software ImageJ (Rasband 1997–2018). The number of hairs was set in relation to the area of the image (hairs mm^−2^; Supp 4a). On the same images, we measured the venation length per leaf area, as an index of the leaf roughness. This index gives information about the unevenness on the leaf surface. Additionally, we took a microscopic photo of a cross-section of each leaf, which included the mid-vein (Supp 4b). We measured the surface length of the cross-section and the leaf width on the image, and used their ratio as a measure of the roughness. Again, we conducted the image analyses with the software ImageJ (Rasband 1997–2018). As an indicator for the flexibility of the leaves, we measured the resistance to punch with a punch force tester (Electric Test Stand TVM-N with dynamometer FH50, Supp 4c). Each leaf was punched three times at different spots between the leaf veins, and we measured the force needed to penetrate the leaf. For measuring the wettability of a leaf at ambient air pressure, we dropped a single tap water droplet of 0.1 mL on each leaf surface (Bartell and Merrill 1932; Räsänen et al. 2013; Yuan and Lee 2013; Supp 4d). Then, we took a lateral photo of the droplet with a normal camera (Nikon D5100, Objective Sigma 18–250 mm F3,5–6,3 DC Macro OS HSM, with super macro conversion lens (DCR-250, Raynox)). On the image, we measured the contact angle of the droplet again with the software ImageJ (Rasband 1997–2018).

### Statistics

All statistical analyses were done with the statistical software R (R Core Team 2017). In our main analysis, we ran a linear mixed effect model to investigate how sediment load on entire leaves depended on the various leaf traits we studied. In this model, sediment load was the response variable, the different leaf traits were treated as fixed factors (see Supp 3) and we included species identity, aquarium, leaf ID and position within the aquarium as random factors. For the final model, we removed leaf position as a random factor, since models including it were not parsimonious (tested using AIC). To fulfil model assumptions regarding the normality of the error distribution, the response variable (amount of sediment per leaf area) was natural log transformed. When traits were highly correlated with each other, we removed those least strongly related to sediment load from initial models to avoid multicollinearity. The traits we removed were hair type and density on the abaxial leaf side and the roughness approximated by vein length. Thus, our initial model contained the following traits: log(area), length, perimeter, pinnation, adaxial hair density, adaxial hair type (category “no hairs” as control hair type), waviness of leaf cross-section, resistance to punch, contact angle and the interaction between log(area) and the adaxial hair density. We constructed our initial model using the ‘lmer’ function in the lme4 library (Bates et al. 2015), and applied a REML fitting procedure. We followed a stepwise procedure to remove fixed factors from the initial model that were not significant (*p* > 0.05), until we could select a final model in which all fixed factors were significantly related to sediment load. For standardizing the regression coefficients we used the ‘beta’ function in the reghelper library (Hughes 2018) and for the coefficients of variation including and excluding the random factors, we used the ‘r.squaredGLMM’ function in the MuMIn library (Barton 2018).

The same procedure was used for the leaves of standardized size. The random factors were the same (species, aquarium, leaf ID and position), while leaf position was, for the same reason as above, removed from further models. Also for this data set, the traits hair type and density on the abaxial leaf side and the roughness measured by vein length were removed due to multicollinearity with other traits. The initial model for the size-standardized leaves then contained the following traits: adaxial hair density, adaxial hair type, waviness of leaf cross-section, resistance to punch and the contact angle.

For the 12 species, we used both in the main experiment with entire leaves and in the experiment with size-standardized leaves, we tested the differences in sedimentation on the leaf surface of each species using paired two-sample *t* tests.

## Results

### Area and pubescence explain sedimentation on entire leaves

Our analysis of entire leaves showed that traits related to total area and pubescence on the upper side were the strongest predictors of sedimentation on the whole leaf surface. In particular, log(area), adaxial hair density, adaxial hair type and the interaction between adaxial hair density and log(area) significantly explained sedimentation (Table 1), and explained a high proportion of its variance (*R*^2^_m_ = 0.65 [variation explained by fixed factors only], *R*^2^_c_ = 0.82 [variation explained by fixed and random factors]).

**Table 1:** Statistical model results of the species sets with entire leaves and size-standardized leaves

	Entire leaves			
	Estimate	Std. error	*t* value	*p* value
(Intercept)	0.044	0.092	0.471	< 2e−16***
Hair type (single hairs) adaxial	0.231	0.109	2.129	0.044*
Hair type (split hairs) adaxial	0.259	0.095	2.714	0.012*
Hair type (felt-like hairs) adaxial	0.033	0.102	0.323	0.750
Hair density (many hairs) adaxial	0.256	0.111	2.316	0.034*
Log area	− 0.629	0.060	− 10.465	< 2e–16***
Interaction hair density (no or few hairs) adaxial and log area	0.212	0.059	3.561	0.001***

	Size-standardized leaves			
	Estimate	Std. error	*t* value	*p* value
(Intercept)	− 0.008	0.192	− 0.042	0.097
Hair density (many hairs) adaxial	0.460	0.171	2.694	0.025*
Waviness of a cross-section	0.398	0.171	2.334	0.045*

Estimates are standardized and *p* value was calculated by type II anova with Kenward–Roger method for the *F* test

**Fig. 2:**
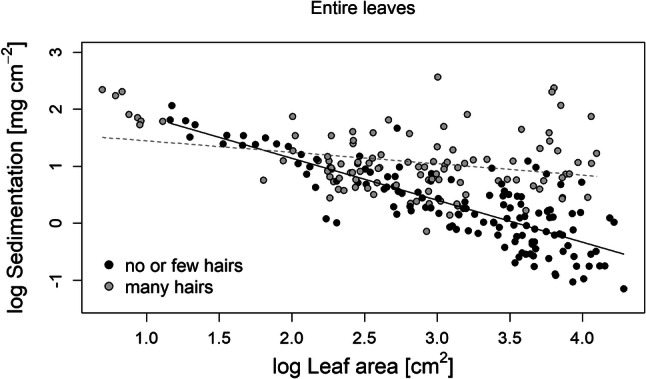
Relationship of the log sedimentation (mg cm^−2^) depending on the log leaf area (cm^2^), for entire leaves. Black dots represent leaves with no or few hairs (< 1 hair mm^−2^; *p* < 0.01; black regression line), and grey dots represent leaves with many hairs (≥ 1 hair mm^−2^; grey dashed trend line)

Sedimentation on the leaf surface decreased with total leaf area, but only on leaves with a low hair density (< 1 hair mm^−2^, *p* < 0.01, Fig. 2). Furthermore, sedimentation was significantly higher on leaves with a high adaxial hair density (≥ 1 hair mm^−2^) compared with leaves with low adaxial hair density (< 1 hair mm^−2^; *p* = 0.03; Supp 5a).

**Fig. 3:**
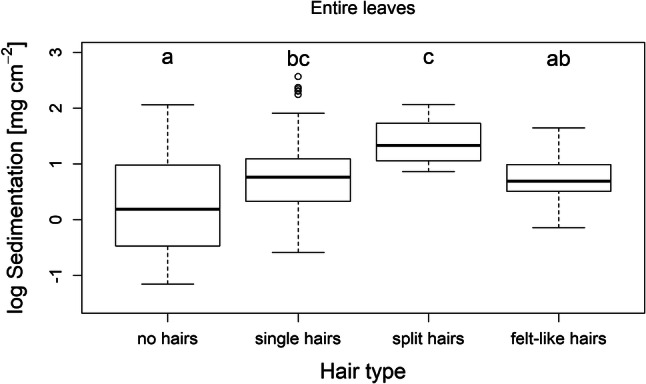
Boxplot of species set with entire leaves showing the significant differences in accumulated sediment per area depending on the hair types. Significance for differences (*p* < 0.05) indicated by letters (a, b, c)

Sedimentation also differed in relation to hair type, where it was lowest on leaves without hairs and highest on leaves with split hairs. Significant differences occurred for “no hairs” compared to “single hairs” and “split hairs”, and for “split hairs” compared to “felt-like hairs” (*p* < 0.05; Fig. 3).

### Pubescence and waviness explain sedimentation on size-standardized leaves

**Fig. 4:**
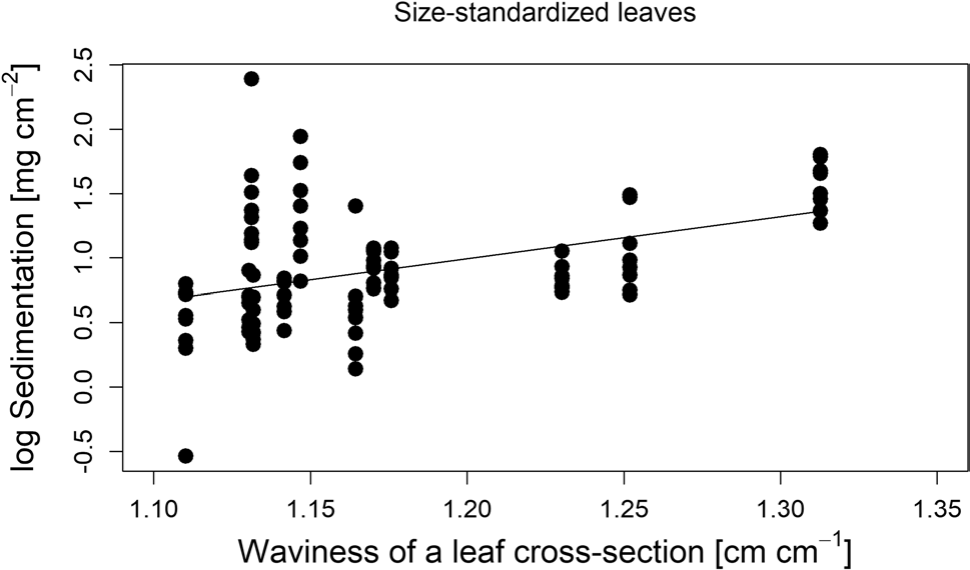
Relationship between the log sedimentation (mg cm^−2^) depending on the waviness of leave cross-sections (cm cm^−1^) for the species set with size-standardized leaves

To investigate which leaf traits are driving sedimentation on size-standardized leaves, we also analysed drivers of sedimentation on leaves cut to a standardized size of 2 × 6 cm^2^. Again, adaxial hair density was an important predictor of leaf sedimentation (*p* = 0.02, Supp 5b). Additionally, we found that increasing waviness of a leaf cross-section (*p* = 0.04, Fig. 4), a variable representing the leaf roughness, increased leaf sedimentation. The proportion of variance explained by the fixed factors was *R*^2^_m_ = 0.36, while random factors also explained a large proportion of variation *R*^2^_c_ = 0.71 (Table 1).

### Within species comparison between entire and size-standardized leaves

**Fig. 5:**
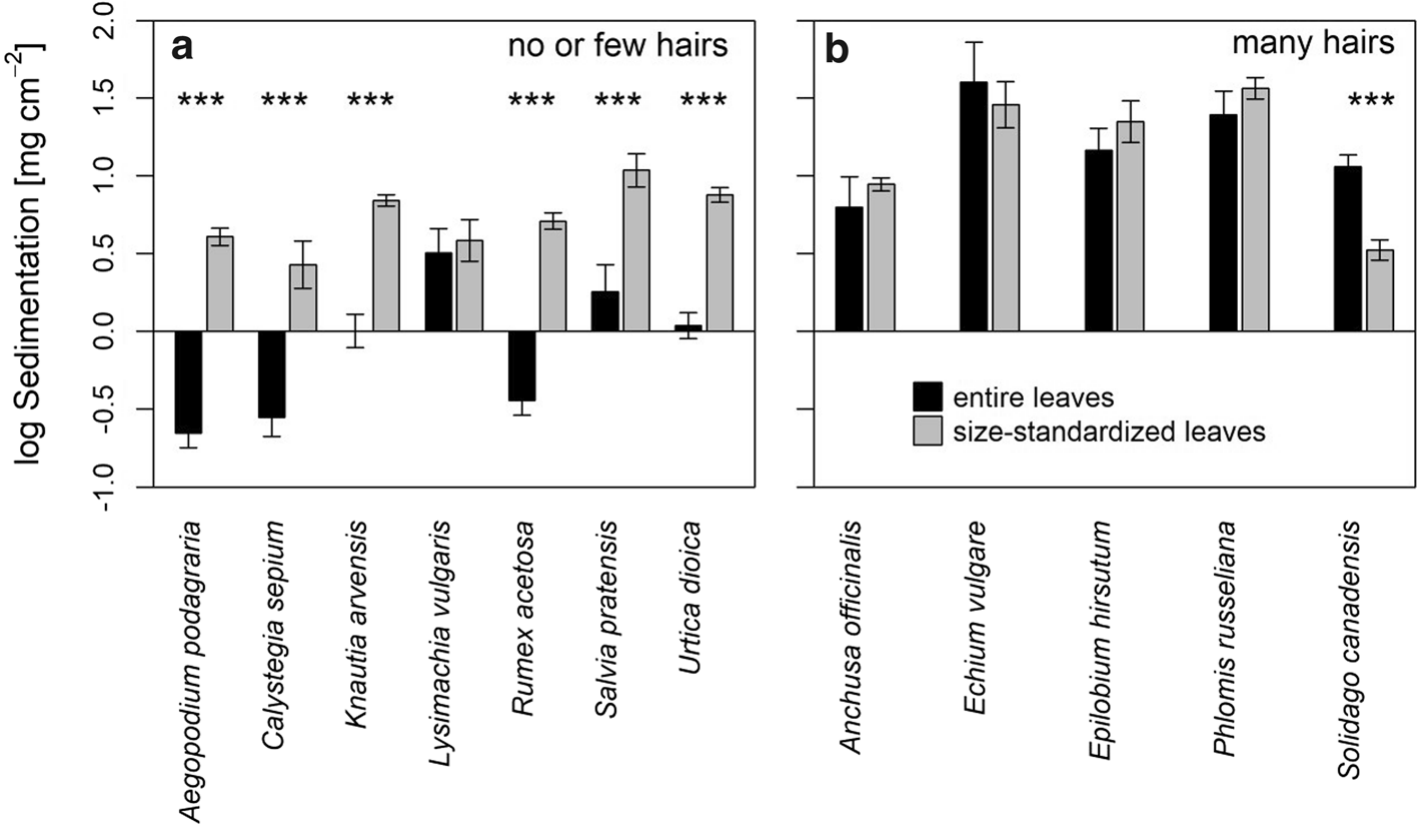
Boxplots of within species comparison between entire leaves and size-standardized leaves, separated due to the hair density **a** no or few hairs, **b** many hairs. Color coded due to the species set

By comparing sedimentation on entire leaves (varying size, 9.44–72.50 cm^2^) with size-standardized leaves (12 cm^2^) from the same species, we could experimentally assess how a standardization of size and form of leaves drives sedimentation per leaf area. This comparison showed that for species with low hair density (< 1 hair mm^*−*2^) the leaf sedimentation per area was higher on size-standardized (i.e. size-reduced in all cases) leaves for six out of seven species (Fig. 5a). In contrast, sedimentation per area is not affected by size-standardization (i.e. size-reduction) for four out of the five hairy species (≥ 1 hair mm^*−*2^; Fig. 5b). The only hairy species with significantly more sediment on entire leaves (*Solidago canadensis*, Fig. 5b), was the species were half of the selected entire leaves were smaller than 12 cm^2^ (9.44–11.63 cm^2^).

These experimental results clearly show the same interaction effect we already observed between the log area and the hair density for the entire leaves (Fig. 2). Sedimentation decreased with increasing area for species with no or few hairs, while there was no significant effect for hairy species.

## Discussion

We showed that leaf traits control the amount of accumulated sediment on leaf surfaces. In our experiment the leaf traits area, pubescence and roughness influence sedimentation significantly, while flexibility and wettability do not. Our results support our hypothesis that sedimentation increases with decreasing leaf area, although only on leaves with low hair density. Length, perimeter and pinnation of the leaves did not influence sedimentation significantly. Furthermore, we could also confirm the hypothesis of increasing sedimentation with increasing adaxial pubescence and roughness, though the latter matters only on size-standardized leaves.

### Area and hair density and their interaction

Our results show that leaf area and pubescence are the strongest drivers of sediment accumulation on leaf surfaces. Previous studies focusing on how airborne particle deposition is related to leaf area have provided mixed results. While Räsänen et al. (2013) found a decrease of particle deposition with increasing leaf area, Sæbø et al. (2012) and Weber et al. (2014) did not find any significant relationship. Our work provided experimental evidence for a negative relationship between sedimentation and leaf area, but only for leaves with low hair density (Figs. 2, 5). The negative area effect for species with low hair density might be driven by the hydrodynamics along the boundary layer of the leaf surface. Within a laminar flow, a boundary layer forms on the leaf surface and becomes thicker with the flow direction (Nepf 2012). At a certain distance along the surface, the boundary layer starts to become turbulent (Nepf 2012). From that distance onwards, turbulence may hinder sediment to settle during moments of high flow velocity, and the turbulence may cause sediment remobilisation from the leaf surface (Nepf 2012). Although in our experiment, we did not have laminar conditions, the sedimentation seems to follow a similar pattern. Small leaves without hairs may be able to accumulate sediment on the surface within a laminar boundary layer, while larger leaves experience turbulence at the distant part of the leaf resulting in less overall sedimentation. To quantify this effect sedimentation needs to be measured on different parts of the leaf surface. On a floodplain meadow, flow velocity varies and turbulence occur within the vegetation patches, caused by structural parameter of the vegetation, such as stem and patch density (Corenblit et al. 2009,2011; Kervroëdan et al. 2018). This may influence sedimentation on leaf surfaces, additionally. Nevertheless, our experiment enables a first understanding of the process of sedimentation on leaf surfaces explained by leaf traits under constant conditions.

Hairs on a leaf surface present obstacles to the water flow. As a consequence, the main drag force of the water is above the hairs (Nepf 2012). With reduced drag force, the flow velocity and turbulence strength between the hairs are reduced (Nepf 2012), which gives the sediment space and time to settle. However, when hair density is low, the few hairs present are not sufficient to significantly alter water flow, leading to similar flow conditions as on plain surfaces (Nepf 2012). This explains the high sedimentation on leaves with many hairs compared to the low sedimentation on leaves with few or no hairs (Supp 5a). In addition, hairs enlarge the surface area of the leaves and thereby the surface for sedimentation. In line with this, previous airborne studies showed that densely haired leaves accumulate more particles (Wedding et al. 1975; Sæbø et al. 2012; Räsänen et al. 2013; Weber et al. 2014). Furthermore, for sedimentation on leaf surfaces with many hairs the effects of the boundary layer are negligible, since the hair layer itself acts as a buffer zone, irrespective of whether the flow above is laminar or turbulent (Paul et al. 2014). Within the hair layer, the flow velocity is reduced and sedimentation takes place (Nepf 2012; Paul et al. 2014).

In our study, we used floodplain species and additional non-floodplain species to span the trait gradients. All species we classified as densely haired prior to the experiment were species that typically grow outside of floodplain areas, but the pubescence of our measured data were classified in two groups (< 1 hair mm^−2^ or ≥ 1 hair mm^−2^). Out of the 13 hairy species, 5 species were floodplain species (*Artemisia vulgaris, Epilobium hirsutum, Lythrum salicaria, Solidago canadensis, and Stachys palustris*). Regarding leaf area, seven species have a below-average leaf area with < 1 hair mm^−2^, of which six are floodplain species (*Calamagrostis epigejos, Convolvulus arvensis, Deschampsia cespitosa, Plantago lanceolate, Saponaria officinalis, Tanacetum vulgare*). This indicates that species with leaf surface traits that are most suitable for leaf sedimentation are well represented among floodplain species. Therefore, management favouring plant species that maximize leaf sedimentation does not require the introduction of exotic species.

### Hair type and waviness of cross-section

Our results also provide evidence that in addition to hair density, the hair type influences surface sedimentation. Leaves without hairs accumulate significantly less sediment than leaves with single hairs or split hairs (Fig. 3). In our study, the category split hairs was represented by only one species (*Phlomis russeliana*). The species has stellate hairs with five ends forming minute canopies, which create a buffer zone against the flow. From forest canopy studies, it is known that wind flow speed is strongly reduced below the canopy (Oliver 1971; Jiao-jun et al. 2004). Again, this may give space and time for sediment to settle (Paul et al. 2014). While we expected a similar effect for species with felt-like hairs, our results do not provide evidence for this. Leaves with felt-like hairs accumulate significantly less sediment than leaves with split hairs. Hairs are differently constructed, with the main purpose to reduce water loss, but they also fulfil defence purposes (Johnson 1975). In our study, the species with split hairs and felt-like hairs do strongly differ in the stiffness, thickness and flexibility of the hairs. The split hairs of *Phlomis russeliana* are stiff, thick and upright, while the felt-like hairs of *Artemisia vulgaris* and *Potentilla anserina* are smooth, thin and flexible. Thus, split hairs are more resistant against the flow and keep standing upright like a forest in the storm, building a canopy underneath which sediment can settle, while felt-like hairs possibly bend with the flow. Also Weber et al. (2014) observed high airborne particle accumulation on species with dense stellate hairs, while Ren et al. (2007) found that species with star-shaped hairs have the strongest cohesive force on the surface. Felt-like hairs still act as obstacle in the flow, however, the accumulated sediment does not significantly differ from leaves with single hairs, or from leaves with no hairs.

When focusing on cut leaves, which had a standardized size and shape, we found that leaf waviness, as a measure of roughness, also significantly increased sedimentation in addition to hair density. Wavier and thus rougher leaves accumulated more sediment, probably through similar mechanisms as those operating on hairy leaves. Strong waves on the leaf provide slipstream areas for sediment to settle. Other studies also found that with increasing leaf roughness the accumulation of airborne particle increases (Little 1977; Pyatt and Haywood 1989; Weber et al. 2014). While our results regarding the effects of leaf waviness are thus in line with our expectations and other studies, we also found that one species, *Echium vulgare*, obtained the highest sediment load despite low waviness values. However, this might represent a limitation of our waviness measurement. The waviness of our cross-sections only indicates the lateral roughness at one cut at a single location per leaf, rather than an overall measure for leaf roughness. *Echium vulgare* has small humps with hairs on the surface, but these were not well represented in our measurements. We would suggest that a 3D scan of the macro-roughness of the leaf surface may better capture the overall leaf roughness, and might be a stronger predictor of leaf surface sedimentation than the waviness of cross-sections that we used in our study.

### Importance of leaf sedimentation for floodplains

The magnitude of variation of the accumulated sediment and the significance and comparatively high proportion of explained variance of the presented results (*R*^2^_m_ = 0.65) indicates the importance of leaf surfaces for sediment accumulation. While this was already shown regarding airborne particle capturing (Sæbø et al. 2012; Räsänen et al. 2013; Weber et al. 2014), to our knowledge, this is the first study on sediment accumulation on the leaf surface of inundated herbaceous species in a setting simulating flood events. Sediment retention is a key ecosystem service provided by floodplain vegetation and our study provides evidence that the traits of the leaves influence the extent to which sedimentation occurs. Elliott (2000) already showed that emergent vegetation within a channel is highly relevant for overall sedimentation within the stream and strongly driven by lateral structural complexity of the stand. Fine sedimentation is in general highest within patches of dense herbaceous vegetation compared to patches of post-pioneer shrubland and forest (Corenblit et al. 2009). In this study, we showed that the functional and structural diversity of the vegetation plays a key role for fine sediment retention on plants. This adds to the growing body of the literature providing evidence for the importance of plant diversity and identity for controlling ecosystem functions (Díaz and Cabido 2001). By combining our understanding of on-plant sedimentation with sedimentation in-between plants in future experiments, we will develop a more holistic picture on the capacity of floodplains to filter sediment from the floodwater. This understanding is crucial for enhancing the ecosystem service of sediment and nutrient retention of floodplains, by guiding restoration projects along rivers (Tockner and Stanford 2002; Palmer et al. 2010). Furthermore, increased insights into on-plant sedimentation may improve the management of already existing floodplain meadows.

## Electronic supplementary material

Below is the link to the electronic supplementary material.

Supplementary file1 (DOCX 420 kb)

## Data Availability

Data will be deposited in the iDiv Data Repository (https://idata.idiv.de) and will get a DOI.
