# Supplementary material for: Leaf area and pubescence drive sedimentation on leaf surfaces during flooding
Source: Oecologia. 2020 May 17;193(3):535–45. doi: 10.1007/s00442-020-04664-2 (PMC7406488; doi:10.1007/s00442-020-04664-2)
Supplement: Supplementary file 1 — Supplementary file1 (DOCX 420 kb) [file 442_2020_4664_MOESM1_ESM.docx]

**Supporting information**

**Supp 1** Selection of species for the experiment was done in such a way as to maximize the variation in the trait expected to be of relevance. ‘Species origin’ implies if the species was inventoried along the river Mulde (“WildeMulde” project) or chosen from the Botanical Garden in Leipzig (BoGa). Size standardized indicates whether the species was, in addition to the main experiment, also present in the experiment in which leaves were cut to standardized size (2x6 cm^2^), which was only possible for sufficiently large leaves.

| **Species** | **Family** | **Species origin** | **Size stand-ardized** | **Pubescence** | **Rough-ness** | **Flexibility** |
| --- | --- | --- | --- | --- | --- | --- |
| *Lysimachia vulgaris* | Primulaceae | Mulde | Yes | Hairless | Smooth | Flexible |
| *Rumex acetosa* | Polygonaceae | Mulde | Yes | Hairless | Smooth | Flexible |
| *Saponaria officinalis* | Caryophyllaceae | Mulde | No | Hairless | Smooth | Flexible |
| *Calystegia sepium* | Convolvulaceae | Mulde | Yes | Hairless | Smooth | Stiff |
| *Convolvulus arvensis* | Convolvulaceae | Mulde | No | Hairless | Smooth | Stiff |
| *Plantago lanceolate* | Plantaginaceae | Mulde | No | Hairless | Smooth | Stiff |
| *Aegopodium podagraria* | Apiaceae | Mulde | Yes | Hairless | Rough | Flexible |
| *Calamagrostis epigejos* | Poaceae | Mulde | No | Hairless | Rough | Flexible |
| *Filipendula ulmaria* | Rosaceae | BoGa | No | Hairless | Rough | Flexible |
| *Deschampsia cespitosa* | Poaceae | Mulde | No | Hairless | Rough | Stiff |
| *Epilobium hirsutum* | Onagraceae | Mulde | Yes | Hairless | Rough | Stiff |
| *Lythrum salicaria* | Lythraceae | Mulde | No | Hairless | Rough | Stiff |
| *Knautia arvensis* | Caprifoliaceae | BoGa | Yes | Sparse hairs | Smooth | Flexible |
| *Tanacetum vulgare* | Asteraceae | Mulde | No | Sparse hairs | Smooth | Flexible |
| *Lupinus polyphyllus* | Fabaceae | BoGa | No | Sparse hairs | Smooth | Stiff |
| *Onobrychis viciifolia* | Fabaceae | BoGa | No | Sparse hairs | Smooth | Stiff |
| *Solidago canadensis* | Asteraceae | Mulde | Yes | Sparse hairs | Smooth | Stiff |
| *Centaurea jacea* | Asteraceae | BoGa | No | Sparse hairs | Rough | Flexible |
| *Geum urbanum* | Rosaceae | BoGa | No | Sparse hairs | Rough | Flexible |
| *Stachys palustris* | Lamiaceae | Mulde | No | Sparse hairs | Rough | Flexible |
| *Artemisia vulgaris* | Asteraceae | Mulde | No | Sparse hairs | Rough | Stiff |
| *Silene latifolia* | Caryophyllaceae | BoGa | No | Sparse hairs | Rough | Stiff |
| *Urtica dioica* | Urticaceae | Mulde | Yes | Sparse hairs | Rough | Stiff |
| *Anchusa officinalis* | Boraginaceae | BoGa | Yes | Dense hairs | Smooth | Flexible |
| *Echium vulgare* | Boraginaceae | BoGa | Yes | Dense hairs | Smooth | Flexible |
| *Phlomis russeliana* | Lamiaceae | BoGa | Yes | Dense hairs | Smooth | Flexible |
| *Geranium molle* | Geraniaceae | BoGa | No | Dense hairs | Rough | Flexible |
| *Lamium maculatum* | Lamiaceae | BoGa | No | Dense hairs | Rough | Flexible |
| *Potentilla anserina* | Rosaceae | BoGa | No | Dense hairs | Rough | Flexible |
| *Salvia pratensis* | Lamiaceae | BoGa | Yes | Dense hairs | Rough | Flexible |


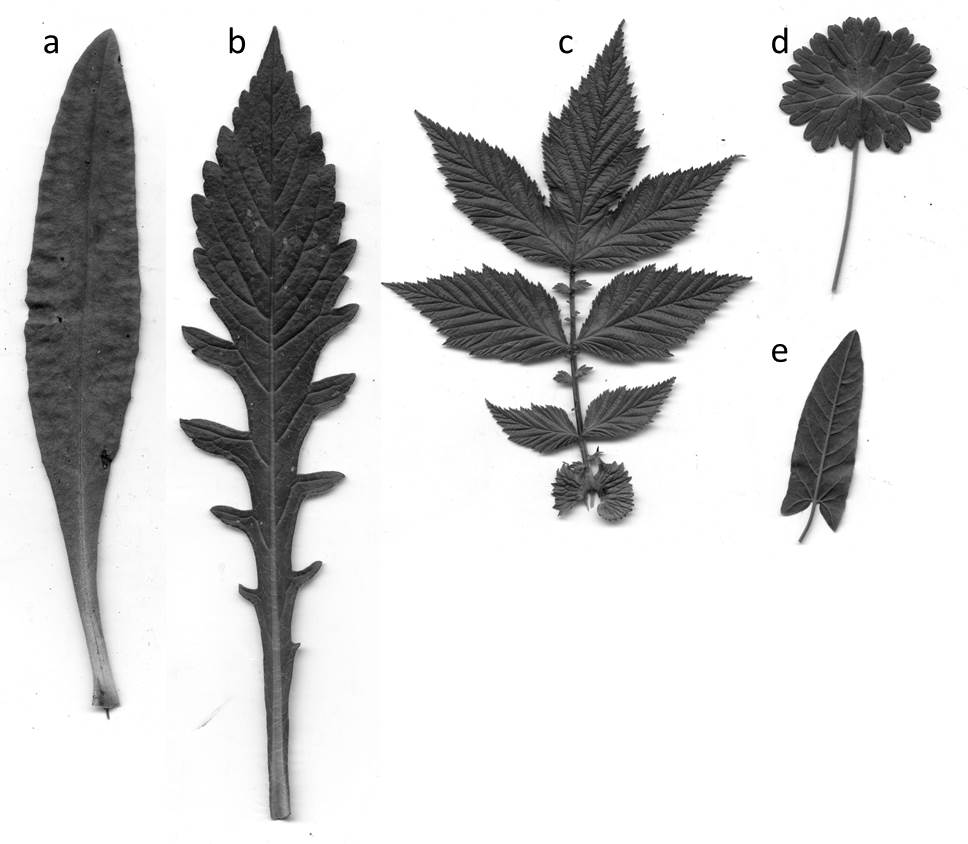


**Supp 2** Scans to measure the leaf morphology (length, area, perimeter and pinnation) of single leaves (a: Anchusa officinalis, b: Knautia arvensis, c: Filipendula ulmaria, d: Geranium molle and e: Convolvulus arvensis).

**Supp 3**  Overview of all measured traits with units and explanation of the categories.

| **Trait** | **Measurement** | **Unit or categories** |
| --- | --- | --- |
| **Leaf morphology** | Length | cm |
|  | Area | cm^2^ |
|  | Perimeter | cm |
|  | Pinnation | Entire, serrated, pinnate, separated |
| **Pubescence** | Adaxial hairs  (upper leaf side) | Little hairs (< 1 hair mm^-2^) |
|  |  | Many hairs (≥ 1 hair mm^-2^) |
|  | Abaxial hairs  (lower leaf side) | Little hairs (< 1 hair mm^-2^) |
|  |  | Many hairs (≥ 1 hair mm^-2^) |
|  | Adaxial hair type  (upper leaf side) | No hairs, single hairs, |
|  |  | split hairs, felt-like hairs |
|  | Adaxial hair type  (lower leaf side) | No hairs, single hairs, |
|  |  | split hairs, felt-like hairs |
| **Roughness** | Roughness  (vein length) | Length of all veins on the leaf per area (cm cm^-2^) |
|  | Waviness of the  leaf cross-section | Surface length of a cross-section per absolute length of the leaf width  (cm cm^-1^) |
| **Flexibility** | Resistance to punch between the veins | N |
| **Wettability** | Negative contact angle  of a water droplet  on the leaf | ° |


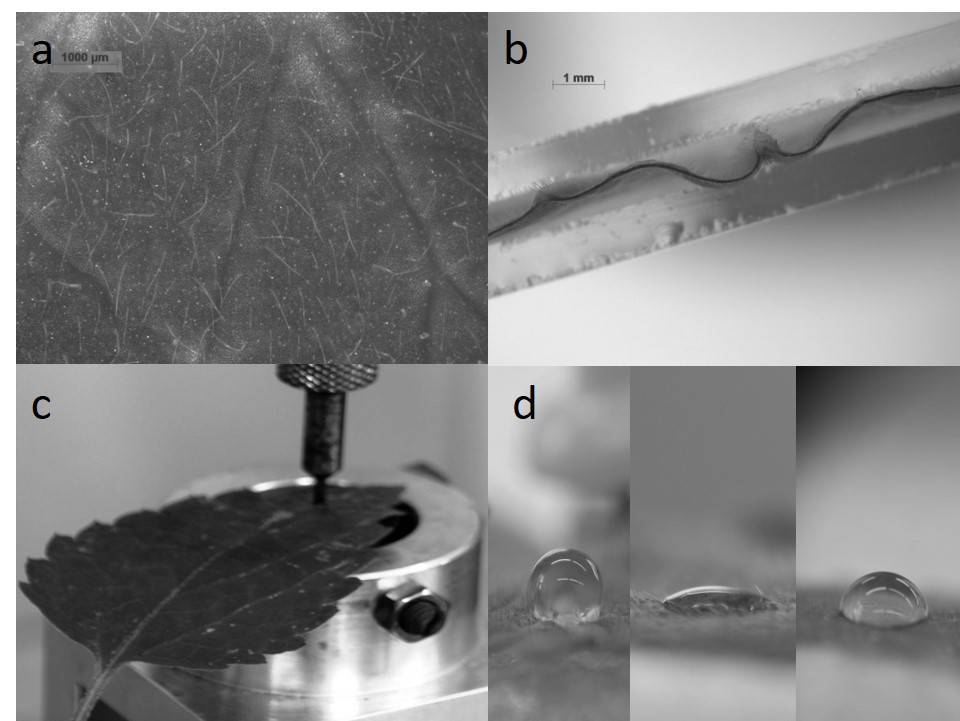


**Supp 4** Images with the microscopic camera of a) a surface of a leaf (*Geranium molle*) and b) the cross-section of a leaf (*Aegopodium podagraria*). c) Resistance to punch test (*Urtica dioica*) and d) Image of water droplets on leaf surfaces to measure the contact angle (from left to right: *Geranium molle*, *Geum urbanum*, *Urtica dioica*).


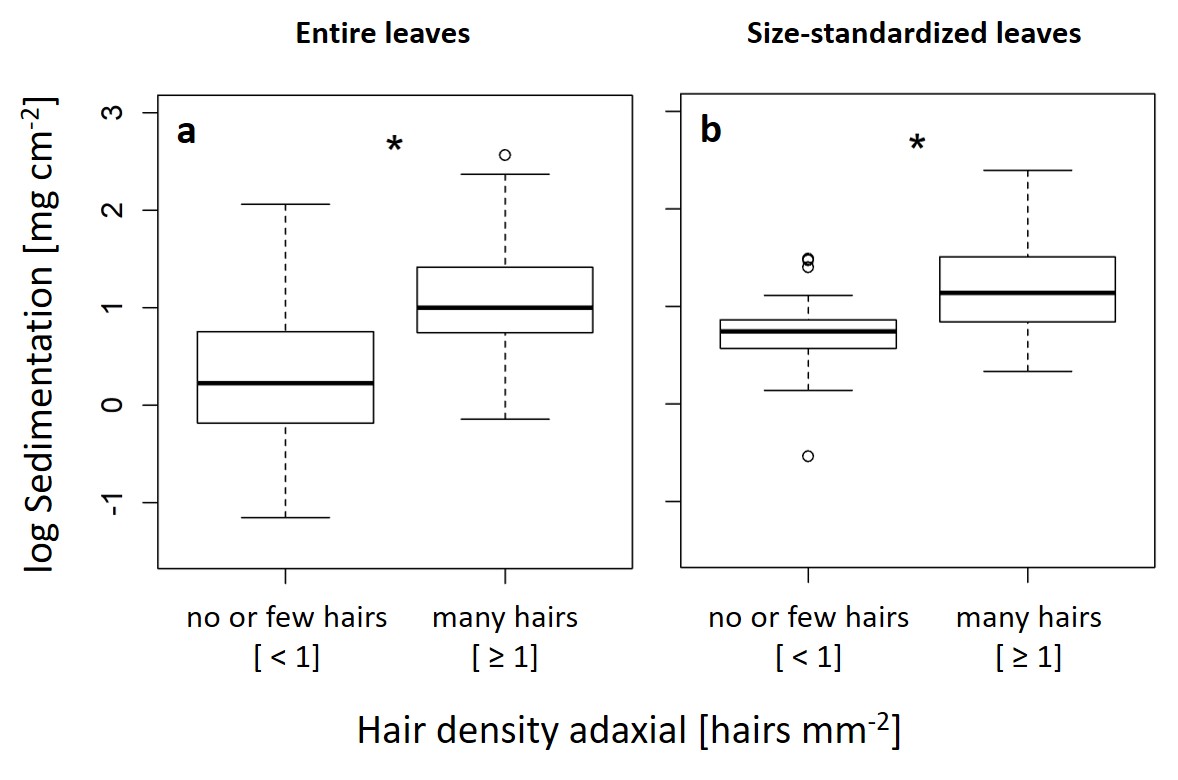


**Supp 5** Boxplots of species sets with (a) entire leaves and (b) size-standardized leaves showing the significant differences in accumulated sediment per area for two groups of adaxial hair density (p=0.034, p=0.025, respectively).


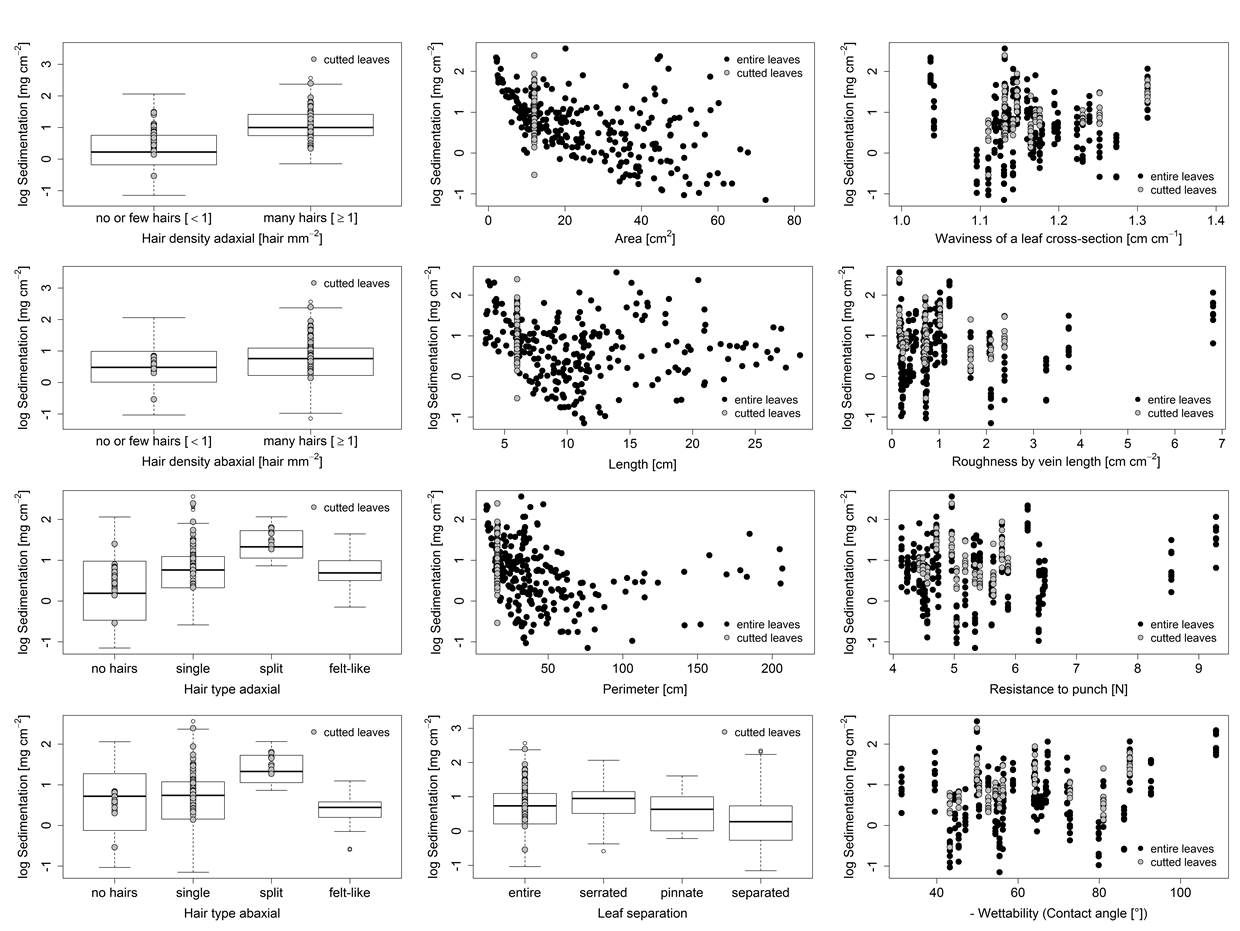


**Supp 6** Graphs of all measured traits and the correlation with the log Sedimentation of the entire species set and the cutted species set.
